# Unveiling the Masquerading of Myocardial Bridging in Cardiovascular Diseases

**DOI:** 10.31083/RCM36868

**Published:** 2025-07-08

**Authors:** Song Wu, Danni Wu, Xianlun Li

**Affiliations:** ^1^China-Japan Friendship Hospital (Institute of Clinical Medical Sciences), Chinese Academy of Medical Sciences & Peking Union Medical College, 100029 Beijing, China; ^2^Department of Integrative Medicine Cardiology, China-Japan Friendship Hospital, 100029 Beijing, China

**Keywords:** myocardial bridging, hypertrophic cardiomyopathy, MINOCA, INOCA, atherosclerosis

## Abstract

Myocardial bridging (MB) is a congenital coronary artery anomaly initially regarded as a benign anatomical variant. However, an increasing number of studies have revealed the association between MB and various cardiovascular diseases. The primary pathological mechanisms underlying the relationship include dynamic mechanical compression leading to myocardial ischemia, coronary vasospasm, and the development of proximal atherosclerosis. Advancement of coronary artery imaging technology has enhanced the understanding of the anatomical and hemodynamic features of MB. Although treatment strategies are primarily symptom-driven, morphological and functional evaluation of MB in patients with asymptomatic concomitant cardiovascular diseases is recommended. Pharmacological therapy and management of cardiovascular conditions are the first-line approach. Invasive treatments strategies should be tailored to individual circumstances. This review examines the relationship between MB and other cardiovascular conditions, such as hypertrophic cardiomyopathy (HCM), coronary atherosclerosis, and myocardial ischemia with non-obstructive coronary arteries (INOCA) or myocardial infarction with non-obstructive coronary arteries (MINOCA). It provides an overview of the underlying mechanisms, diagnostic assessments, and treatment strategies. However, large-scale randomized controlled trials are needed to validate these findings.

## 1. Introduction

Myocardial bridging (MB), a congenital coronary anomaly first documented by Reyman in 1737 [1], occurs when a segment of coronary artery tunnels through myocardial tissue. The myocardial fibers and the intramyocardial segment are referred to as the “myocardial bridge” and “tunneled artery”, respectively. Subsequently, Postman reported the phenomenon of “milking”, where the tunneled segment narrows during systole, distinguishing MB from organic stenosis [2]. The prevalence of MB varies significantly across studies, primarily due to differences in diagnostic methods. The reported prevalence rates are 21.7% for coronary computed tomography angiography (CCTA), 4.3% for conventional angiography, 44.6% for autopsy/cadaveric dissection, 71.5% for stress echocardiography, and 22.7% for intravascular ultrasound [3]. Furthermore, MB prevalence may be influenced by gender and population differences [3, 4]. The left anterior descending (LAD) coronary artery, particularly its middle segment, is the most common site for MB [3]. The progress in both invasive and non-invasive coronary imaging techniques and functional testing has significantly improved our understanding of MB pathophysiology and refined the management of MB [5]. Although MB was initially considered a benign anatomical variation, a meta-analysis has shown the association between MB and major adverse cardiac events, especially myocardial ischemia [6]. Currently, pharmacological therapy remains the first-line treatment for symptomatic patients, whilst interventional and surgical approaches are recommended for patients who are refractory to pharmacological treatment [7]. Invasive strategies should be guided by factors such as the effectiveness of medication, the morphological and functional characteristics of MB, and patient preferences. Patients with symptomatic isolated MB generally have a good long-term prognosis with appropriate treatment [8].

However, an increasing body of research indicates that MB may contribute to the progression of concomitant cardiovascular diseases (CVDs), leading to adverse prognoses [5, 9, 10]. Compared to isolated MB, the prevalence of MB is higher in patients with hypertrophic cardiomyopathy (HCM), suggesting a potential association between the two [11]. The relationship between MB and atherosclerosis is complex, and its role in the development of atherosclerosis remains controversial [12, 13, 14]. With the concepts of myocardial infarction with non-obstructive coronary arteries (MINOCA) and ischemia with non-obstructive coronary arteries (INOCA) raised, MB-related non-obstructive coronary artery disease (NOCAD) has gradually gained recognition [10, 15]. A thorough understanding of the pathophysiological relationship between MB and concomitant CVDs is crucial for more comprehensive and effective management of MB. This review focuses on the clinical relevance of MB in the context of other CVDs, with particular emphasis on HCM, coronary atherosclerotic heart disease, and MINOCA or INOCA.

## 2. Pathophysiology of MB-Related Myocardial Ischemia 

The predominant mechanism remains mechanical compression, although advancements in coronary imaging and functional testing have gradually revealed the multifactorial mechanisms underlying myocardial ischemia in MB. Myocardial filling depends on diastolic flow, while narrowing of the MB occurs during systole [16]. This suggests that MB does not severely impair myocardial perfusion and is typically considered a harmless condition. However, the dynamic compression of MB, which fundamentally distinguishes it from fixed coronary stenosis, can have complex effects on hemodynamics. During periods of increased heart rate, myocardial perfusion becomes increasingly dependent on the blood flow during systole with the diastole shortening. In symptomatic patients, compression of the bridged segment may extend into diastole, impairing coronary perfusion [17, 18]. In addition to elevated heart rate, heightened sympathetic nervous activity can increase myocardial contractility, further exacerbating MB compression and worsening the mismatch between myocardial oxygen supply and demand [16].

It is noteworthy that, in addition to the direct effects of mechanical compression, long-term influences on the tunneled artery also exist. Coronary vasospasm is more common in patients with MB, likely due to microvascular endothelial dysfunction and impaired nitric oxide metabolism resulting from abnormal flow dynamics [19]. Additionally, atherosclerotic plaques are more frequently found at the segment proximal to the MB due to dynamic retrograde flow [7]. Another important phenomenon is “branch steal”, which occurs due to the Venturi effect or viscous pressure loss at the entrance and stenotic segment [16].

Although MB is a congenital condition, symptoms typically manifest in individuals during their thirties or forties [5]. This suggests that aging may play a role in the development of myocardial ischemia in patients with MB. When left ventricular diastolic dysfunction and other comorbidities compromise coronary perfusion during diastole, the impact of MB on reducing coronary reserve capacity becomes more pronounced. Additionally, the depth, length, and location of MB all affect the occurrence of myocardial ischemia [7].

## 3. The Role of CCTA and Cardiac Magnetic Resonance (CMR) in Evaluating MB

Although the initial assessment of MB was performed using coronary angiography, with the widespread application of CCTA in outpatient chest pain evaluations, the use of CCTA in MB has rapidly increased. CCTA offers the advantage of non-invasive assessment, and its high spatial resolution allows for clear visualization of MB morphology and surrounding structures [20]. Furthermore, CCTA can classify the course of the artery as normal (within the epicardial fat), superficial intramyocardial, or deep intramyocardial based on its depth. This classification contributes to guide invasive strategies. Percutaneous coronary intervention (PCI) or myotomy may be suitable for normal or superficial MB, while coronary artery bypass grafting (CABG) might be recommended for very deep (≥5 mm) or long (≥25 mm) MB [7]. CCTA can also assess the degree of MB stenosis and the fat attenuation index (FAI), both of which are related to adverse events [21]. This makes CCTA advantageous for identifying high-risk populations in MB and guiding subsequent early management. With the advancement of computational fluid dynamics, computed tomography-derived fractional flow reserve (CT-FFR) offers a more detailed assessment of the impact of MB on coronary blood flow [22, 23], though further validation is still needed. CCTA is also commonly used to evaluate atherosclerotic plaques, making it particularly suitable for assessing MB in conjunction with coronary atherosclerosis. Additionally, CCTA can be used to calculate endothelial shear stress (ESS), which is valuable for the understanding of MB-related atherosclerosis and coronary spasm [24, 25]. CMR is less commonly used for MB compared to CCTA. Although CMR can visualize the spatial structure of MB, its lower spatial resolution limits its utility. Currently, CMR is primarily used in the evaluation of MB associated with hypertrophic cardiomyopathy, facilitating the simultaneous assessment of myocardial fibrosis [26].

## 4. The Relationship Between MB and Concomitant CVDs

### 4.1 HCM and MB

HCM is a common inherited myocardial disease marked by left ventricular hypertrophy, with or without left ventricular outflow tract (LVOT) obstruction [27]. MB is more frequently observed in HCM patients than in those without it, with prevalence estimates ranging from 11% to 50% [9, 11, 28]. MB most commonly occurs in the LAD, which courses along the interventricular groove and supplies blood to part of the interventricular septum. Since septal hypertrophy is a hallmark of HCM, this anatomical feature may predispose individuals with HCM to the development of MB.

The primary pathological mechanism linking HCM and MB is myocardial ischemia. Even in the presence of structurally normal coronary arteries, myocardial ischemia remains a key pathological feature of HCM. This ischemia can be attributed to several factors, including a supply/demand mismatch, high intraventricular pressure caused by LVOT obstruction, elevated left ventricular end-diastolic filling pressure and compression of the microvasculature due to myocardial hypertrophy, microcirculatory remodeling, and microvascular dysfunction [29, 30]. Given that myocardial ischemia in MB results from mechanical compression, it is reasonable to hypothesize that diastolic delay in MB could be exacerbated by LVOT obstruction and impaired diastolic function in HCM. This hypothesis is further supported by three-dimensional (3D) reconstruction models of the LAD derived from two-dimensional (2D) angiographic imaging, which demonstrate that MB can significantly disrupt diastolic flow patterns in patients with HCM [31].

The role of MB in the prognosis of HCM has been widely debated in recent decades, with differing opinions in both adult and pediatric populations [9, 32]. Zhu *et al*. [33] analyzed a large population sample, excluding patients with HCM, and concluded that MB is associated with adverse cardiac events. However, a recent meta-analysis, which included 10 observational studies, found no association between MB and cardiovascular mortality or nonfatal adverse cardiac events in HCM, except for myocardial ischemia (*p* = 0.04) [9]. This finding should be interpreted with caution due to limitations of the research, including the small sample size, variability in MB definitions, and the complex relationship between ischemic effects and adverse events. Furthermore, MB has been shown to increase the incidence of arrhythmias in patients with HCM, including atrial fibrillation and fatal ventricular arrhythmias, which are the leading causes of death in HCM [28, 34]. Histologically and via CMR imaging, MB has been closely linked to myocardial fibrosis in HCM [35, 36]. Fibrosis, in turn, serves as an important substrate for the development of arrhythmias. Therefore, it is reasonable to conclude that MB exacerbates ischemia in HCM, leading to fibrosis and the subsequent development of arrhythmias.

Currently, the treatment of MB is primarily symptom-driven. However, in patients with MB and HCM, treatment should also focus on preventing adverse events. For symptomatic MB in HCM, pharmacological therapy remains the first-line treatment. Beta-blockers are recommended as the first-line pharmacological choice for MB in HCM. The effect of beta-blockers on heart rate reduction can increase ventricular diastolic time, leading to improved myocardial perfusion, and alleviation of the LVOT obstruction. For patients with malignant arrhythmias in HCM, implantable cardioverter-defibrillators (ICDs) remain the primary therapeutic option [27]. However, case reports have shown that even after ICD implantation, patients with MB and HCM may still require interventional or surgical treatment due to persistent adverse events [37, 38]. This suggests that interventional or surgical treatments should be considered earlier in these patients. PCI has been used for coronary revascularization in MB, but it is associated with a higher incidence of early in-stent restenosis and perforation, raising concerns about long-term outcomes [7]. Although patients with HCM and MB face more post-PCI complications [39], there is still a lack of robust clinical evidence on this matter. Future-generation stents, designed to withstand radial stress from cyclical systolic compression, may be required for these patients. Concomitant CABG during ventricular septal myectomy has been associated with good long-term outcomes in patients with MB and HCM [40, 41]. When choosing grafts for CABG, saphenous vein grafts tend to have a higher patency rate compared to the left internal mammary artery [41]. Supra-arterial myotomy, also known as “unroofing”, is particularly effective when performed on patients with favorable anatomy (non-tortuous arteries, shorter and more superficial intramyocardial courses) and can be safely performed with ventricular septal myectomy [41, 42]. Additionally, robotic technology, with its advantages in visual enhancement and management of postoperative complications, has facilitated the application of unroofing in patients with MB and HCM [43].

The myocardial ischemic effect of MB is amplified by HCM, which in turn promotes the progression of myocardial fibrosis in HCM. Compared with isolated MB, MB in HCM should be treated with caution. The management of MB in HCM faces two major challenges: identifying patients who require further intervention and determining the appropriate treatment strategies. A symptom-guided management strategy may not be suitable for MB in HCM, because the symptoms caused by MB can be obscured by the symptoms of HCM itself. Moreover, asymptomatic patients should not be overlooked due to the pathological link between MB and HCM. The assessment of the morphological and functional characteristics of MB through imaging modalities provides insights for MB patients with HCM who may be at risk of adverse clinical outcomes [31, 36]. Pharmacological therapy remains the first choice. For the occurrence of malignant arrhythmias, ICD implantation can be performed, especially in adolescents [38]. The complications associated with PCI are concerning, and high radial force second generation drug eluting stents may represent a better option [44]. Surgical intervention can be combined with septal myectomy. Both CABG and myotomy can achieve good prognoses [40, 41]. However, the effect of current surgical strategies still needs further refinement through randomized controlled trials (Table 1, Ref. [36, 40, 41, 42]).

**Table 1.** **The effect of different surgical strategy in patients with HCM and MB**.

Study (years)	Study design	Group	Surgical strategy	Median follow-up (years)	Endpoint events	Significant results
Song *et al*. [36] (2024)	Monocenter, Retrospective	HCM+MB (n = 76)	Myectomy+CABG (n = 37)	2.3	All-cause mortality	No difference in all-cause mortality
		HCM alone (n = 416)	Myectomy+Myotomy (n = 34)		Cardiovascular mortality	(*p* = 0.63) or cardiovascular mortality
						(*p* = 0.72) between patients undergoing MB-related surgery and those without MB
Lu *et al*. [40] (2024)	Monocenter, Retrospective	HCM+CAD (n = 223)	Myectomy+CABG	5.1	All-cause mortality	Cumulative survival rates
		HCM+MB (n = 90)				HCM+CAD: 96.2%
		HCM+CAD+MB (n = 7)				HCM+MB: 97.3%
						(*p* = 0.537)
Wang *et al*. [41] (2021)	Monocenter, Retrospective	HCM+MB (n = 203)	Myectomy+CABG (n = 90)	3	All-cause mortality	Cumulative survival rate of all‐cause
		HCM alone (n = 620)	Myectomy+Myotomy (n = 52)		Cardiovascular mortality	mortality (*p* = 0.89) and cardiovascular mortality (*p* = 0.63) were similar among groups
			Myectomy+MB untreated (n = 61)		Nonfatal MI	
					The combined endpoints	Cumulative survival rate of nonfatal MI (*p * < 0.001) and combined endpoints (*p* = 0.02) were significantly lower in the no‐treatment MB group
Kunkala *et al*. [42] (2014)	Monocenter, Case-controlled	HCM+MB (n = 36)	Myectomy+Myotomy (n = 13)	10	All-cause mortality	Cumulative survival rates
			Myectomy+MB untreated (n = 10)			Myectomy+Myotomy: 83.3%
			No surgery (n = 13)			Myectomy+MB untreated: 100%
						No surgery: 67.9% (*p* = 0.297)

MB, myocardial bridging; HCM, hypertrophic cardiomyopathy; CAD, coronary artery disease; CABG, coronary artery bypass grafting; MI, myocardial infarction.

### 4.2 Coronary Atherosclerosis and MB

The formation of atherosclerotic plaques in the coronary arteries is a key pathophysiological process underlying clinical atherosclerotic CVDs, one of the leading causes of death worldwide [45]. The atherosclerosis in MB has long been a topic of concern, but the relationship between the two has remained controversial. Although continuous studies have shown that atherosclerotic plaques predominantly develop in the proximal segment of the MB [12, 21], other research has found the distribution of proximal plaques between patients with MB and those without is comparable [13, 46]. Similarly, while the bridge segment is generally considered to be spared of atherosclerosis, some studies have reported the occurrence of atherosclerotic lesions in this region [13, 47]. It has been proposed that the anatomical structure of the MB contributes to plaque instability in the proximal segment, predisposing individuals to acute coronary syndrome [48, 49]. However, recent studies have indicated that the coronary atherosclerotic burden in patients with MB is comparable to that in patients without MB [50], and even the presence of MB may be a protective factor against atherosclerosis burden [14, 51, 52]. The relationship between MB and atherosclerosis requires careful analysis and further exploration, as most of these findings are derived from retrospective studies, which are susceptible to confounding factors.

The mechanism underlying the predisposition to atherosclerosis in the proximal segment of MB, while the bridged segment remains largely unaffected, has long been a focus of research. Understanding this phenomenon may provide deeper insights into the pathogenesis of atherosclerosis and inspire novel preventive strategies. The coronary arteries undergo morphological changes during the cardiac cycle, leading to alterations in hemodynamics, such as flow velocity and pressure gradients, which ultimately facilitate plaque formation [53, 54]. Ge *et al*. [55] reported that retrograde flow caused by systolic mechanical compression of MB can be observed in the proximal segment using Doppler ultrasound. This results in the abrupt breakage of the propagating forward systolic wave, creating an area of low ESS that plays a key role in the development of atherosclerosis [56]. Low ESS is associated with endothelial dysfunction, and the microenvironment in the proximal segment of MB, influenced by vasoactive substances, contributes to the initiation and progression of atherosclerosis [57]. However, recent research has found that MB is characterized by ESS homogeneity, with local ESS around the entrance of MB remaining stable as assessed by CCTA [24]. It is essential to clarify the differences in ESS assessment across various modalities. Moreover, it has been reported that the pressure within the proximal segment of the coronary artery in MB is higher than aortic pressure, which may increase the risk of endothelial injury [58].

Another intriguing feature of MB is the relatively low incidence of plaques within the tunneled segment of the artery. From an anatomical perspective, this segment is separated from epicardial adipose tissue, which is linked to pro-inflammatory signaling pathways in the surrounding vasculature [59]. Imaging studies support this observation, showing that peri-coronary adipose tissue attenuation, associated with inflammation, is lower in the bridged segment compared to the non-bridged segment, which exhibits a higher atherosclerotic burden [60]. Additionally, the lack of a vascular network in the adventitia of the bridged segment, as shown by optical coherence tomography, limits the interaction between the surrounding adipose tissue and the coronary arteries [61]. Mechanical compression may promote lymphatic drainage, raising the possibility that an atherosclerosis-brain circuit, mediated by neuroimmune cardiovascular interfaces, could play a role [62, 63]. Furthermore, the homogeneous and higher ESS in the bridge segment, cause the endothelial cells beneath the MB to become spindle-shaped and regularly engorged along the direction of blood flow [24, 64]. The thinner intima and absence of foam cells in the tunneled artery may also contribute to this protective effect against atherosclerosis histologically [52, 65, 66].

Based on current research, we can hypothesize that MB facilitate the progression of proximal atherosclerosis while inhibiting atherosclerosis in the bridge segment. On one hand, the compression of MB can induce hemodynamic changes in the proximal coronary arteries, leading to endothelial dysfunction, which provides a theoretical basis for the development of proximal atherosclerosis [55, 56, 57]. On the other hand, CCTA studies have emphasized the correlation between the degree of MB compression and the extent of proximal atherosclerosis [21, 67]. Moreover, the protective effect of the bridge segment does not imply that no plaques exist within it, which is understandable, as traditional atherosclerotic risk factors may outweigh the protective effect of the bridge segment [68]. Additionally, the anatomical structure of the bridge segment, the hemodynamic alterations induced by mechanical compression, and the pathological changes in endothelial cells support this protective role [24, 52, 59, 60, 61, 62, 63, 64, 65, 66]. Regarding the protective effect of MB on overall atherosclerotic burden, although it may be related to the release of anticoagulants and growth factors induced by MB compression, there remains a lack of robust theoretical support [66]. Large-scale statistical studies have found that this protective effect is more pronounced in patients with advanced atherosclerosis [14, 51], suggesting the hypothesis that the local protective effect of the bridge segment is amplified rather than providing an overall protective effect. Therefore, we conservatively conclude that MB may not exacerbate the overall atherosclerotic burden, and the broader protective effect still requires further mechanistic validation.

While large-scale clinical studies on the treatment of coronary atherosclerosis in MB are lacking, antiplatelet agents and statins are currently in clinical use [69]. Although there is no consensus on interventional treatment strategies for this population, PCI and CABG can be considered based on plaque characteristics. Hao *et al*. [70] reported that severe atherosclerosis in the proximal segment of a myocardial bridge, when treated with PCI, is associated with an increased risk of in-stent restenosis. In this context, early identification of MB patients at risk for atherosclerosis, followed by timely intervention, is crucial in clinical practice. Studies have shown that the length, depth, and location of MB, as well as the myocardial bridge index (MBI, calculated as MB length × MB depth), are associated with an increased risk of proximal plaque formation [53, 71, 72, 73]. However, the clinical application of these findings is limited by various modalities and a lack of validation. Recently, there have been advancements in developing a vascular radiomics model based on CCTA to predict plaque development in the proximal segment of MB [74]. This model effectively integrates anatomical features with hemodynamic changes, enabling more accurate early prevention strategies.

In conclusion, it can be hypothesized that MB promote the development of proximal atherosclerosis while protecting the bridge segment, without increasing the overall atherosclerotic burden in the coronary arteries. For patients with MB combined with proximal atherosclerosis, early control of high-risk atherosclerotic factors and pharmacological treatment are strongly recommended. For those requiring surgical intervention, CABG may be a more viable option than PCI. However, these findings urgently require further validation through randomized controlled trials.

### 4.3 NOCAD and MB

With the advancement of imaging techniques, particularly CCTA and intravascular angiography, NOCAD has received increasing attention. This includes conditions such as INOCA and MINOCA. It is estimated that MINOCA accounts for approximately 6%–8% of myocardial infarction cases, while about half of patients undergoing coronary angiography are diagnosed with INOCA [75, 76]. A large-scale retrospective study suggests that MB is an independent risk factor for MINOCA, with patients having a 3.2-fold higher likelihood of MB compared to those with obstructive coronary artery disease [10]. Additionally, MB is observed in nearly 15.8% of INOCA patients [77]. Studies also have shown that both INOCA and MINOCA are positively correlated with MB [10, 78]. Therefore, MB has increasingly been recognized as a contributing factor in both MINOCA and INOCA.

Coronary vasospasm, a reversible constriction of the epicardial coronary arteries leading to partial or complete occlusion, is another potential underlying mechanism of MINOCA and INOCA [19, 79, 80, 81]. Mechanical compression due to MB may impair endothelial-dependent vasodilation, increasing vascular reactivity to vasoconstrictors [82, 83]. Furthermore, alterations in local ESS within the MB segment, along with an imbalance in vasoactive substances, are also implicated in vasospasm [57]. Vasospasm in the MB segment may be attributed to the thinner intima and higher proportion of smooth muscle cells, leading to a more pronounced vasoconstrictive response [64, 84]. Additionally, spontaneous coronary dissection may serve as a potential mechanistic link between MB and MINOCA [75, 85].

The acetylcholine (Ach) provocation test can induce coronary spasm and is a fundamental method for evaluating coronary vascular reactivity [86]. As the prognosis of MB is associated with a positive test result [19], the Ach provocation test can help guide risk stratification in MB patients. However, clinical application of the Ach provocation test is limited by concerns over potential complications, which can result in misdiagnosis and inappropriate medication use [87, 88]. A meta-analysis on the safety of the Ach provocation test revealed that the overall complication rate was only 0.5%, demonstrating its relative safety [89]. In clinical practice, we can minimize complications by focusing on three key aspects: (1) Selecting appropriate candidates for the test. The specificity of the Acute presentation, Bridge, CRP, and Dyslipidaemia (ABCD) score in identifying patients likely to respond positively to intracoronary Ach provocation testing may improve its accurate application [90]. (2) Before the test, temporary pacing electrodes could be installed, especially for patients who need to have Ach injected into the right coronary artery, which may help avoid the occurrence of severe bradycardia or cardiac arrest [91]. Although 20 µg is currently commonly used as the starting dose, 10 µg may be more favorable for preventing complications in patients with severe vasospasm [91]. The injection speed of Ach is recommended to be over 20 seconds, and up to 3 minutes is also acceptable, especially when the maximum dose of 100 µg is used [89]. (3) The test process requires close monitoring to adjust the test at any time. In the event of serious adverse effects, nitrate drugs can be injected promptly, along with treatment for hypotension or serious arrhythmias.

Long-acting non-dihydropyridine calcium channel blockers (CCBs) are considered the most suitable treatment for MB patients with concomitant vasospasm, as they can alleviate both MB-related compression and vascular spasm [91]. Dihydropyridine CCBs and nitrates are not recommended, as they may exacerbate the systolic compression of MB. It has been found that nitrates used to treat coronary artery spasm associated MB not only fail to improve patient prognosis but also increase the recurrence of symptoms [92]. Although beta-blockers are the first-line treatment for MB, they may potentially induce vasospasm by stimulating α-receptors, which can exacerbate angina symptoms [93]. CABG and unroofing may be considered for patients with MB and vasospasm who do not respond well to pharmacological treatment, though further clinical studies are needed to confirm their efficacy.

In summary, MB is a key etiological factor in NOCAD, with vasospasm serving as the primary pathological mechanism leading to adverse events. The Ach provocation test is useful for the early identification of MB patients who require targeted treatment. For patients with NOCAD and MB, it is recommended to control the risk factors for coronary spasm, such as smoking cessation and combined pharmacotherapy, with non-dihydropyridine CCBs being the first-line choice. ICD may be considered for patients with recurrent malignant arrhythmia or syncope [94]. The effectiveness of surgical interventions still requires further investigation.

## 5. Conclusion

MB is a relatively common congenital coronary artery anomaly encountered in clinical practice. Once considered benign, it is now recognized as being closely linked to various other cardiovascular conditions. The primary shared pathological mechanisms include myocardial ischemia, coronary vasospasm, and atherosclerosis. MB plays a significant role in conditions such as HCM, coronary artery atherosclerotic heart disease, and MINOCA/INOCA. For this specific cohort of MB patients, a comprehensive evaluation of its impact on prognosis is essential. Using pharmacological therapy as an early management strategy is recommended to control these cardiovascular conditions. Invasive strategies should be individualized according to the patient’s specific presentation. However, further randomized controlled trials are required to explore this more thoroughly.

## Declaration of Al and Al-Assisted Technologies in the Writing Process

We would like to acknowledge the use of ChatGPT-4.0 for assisting in grammar and spelling corrections in this manuscript. Its support helped improve the clarity and readability of our work.
